# Is there prejudice from thin air? Replicating the effect of emotion on automatic intergroup attitudes

**DOI:** 10.1186/s40359-020-00414-4

**Published:** 2020-05-06

**Authors:** Junhua Dang, Zeynep E. Ekim, Sarah Ohlsson, Helgi B. Schiöth

**Affiliations:** 1 Department of Neuroscience, Faculty of Medicine, Uppsala University, Uppsala, Sweden; 2 Department of Psychology, Faculty of Social Science, Lund University, Lund, Sweden; 3 Institute for Translational Medicine and Biotechnology, Sechenov First Moscow State Medical University, Moscow, Russia

**Keywords:** Emotion, Prejudice, IAT, Ingroup favoritism, Outgroup derogation

## Abstract

**Background:**

Previous studies showed that anger, rather than sadness, created automatic intergroup bias in a minimal group context.

**Methods:**

The current research reports a single study (*N* = 99) aiming to replicate this finding and further to test whether the intergroup bias manifests as ingroup favoritism, outgroup derogation, or both.

**Results:**

Our results failed to replicate the effect of anger on automatic bias. Intriguingly, participants across all emotion conditions exhibited high level of ingroup favoritism, but there was little evidence of outgroup derogation.

**Conclusion:**

These results suggest that, when there is no competition or conflict between groups, individuals, even in a bad emotional state such as anger, generally show ingroup love rather than outgroup hate.

## Background

Emotions, even those that are incidentally induced by irrelevant stimuli, have substantial influence on people’s cognition, judgment, and behavior [16, 17]. In the research field of stereotype and prejudice, discrete emotions, such as anger and sadness, have been shown to exert different impacts on stereotypic information processing and intergroup attitudes, no matter these impacts were measured explicitly (e.g., [2, 4, 13]) or implicitly (e.g., [1, 14]).

Although most of these studies were conducted in real contexts in which meaningful groups (e.g., Whites and Blacks) were contrasted or evaluated, some researchers claimed that prejudice or intergroup bias could even come from “thin air” [7]. That is to say, emotions have the potential to affect intergroup cognitions and behaviors even when groups are minimally defined by random categorization in the laboratory and thus devoid of cultural or historical meaning (i.e., the minimal group paradigm). In two experiments, DeSteno and colleagues showed that incidentally induced anger in such minimal group context created automatic intergroup bias as indicated by reaction times on the evaluative priming task and the implicit association test (IAT), whereas sadness did not lead to such bias, compared with the neutral emotion [7]. This occurred, as interpreted by these authors, because anger is functionally relevant to intergroup conflict and competition whereas sadness has rather low relevance to intergroup relations.

Despite their inspiring role for following research, these results have never been verified by other research teams. Considering the recent replicability issue in psychology (e.g., [18, 19]), it is important to test whether this seemingly “surprising” finding would be replicated. Further, although DeSteno et al. [7] hypothesized that anger would lead to higher prejudice towards outgroups, actually the tasks they used (i.e., the evaluative priming task and the IAT) cannot differentiate between outgroup derogation and ingroup favoritism [5]. That is to say, the intergroup bias they found may reflect a positive attitude toward ingroups, a negative attitude toward outgroups, or both. Indeed, this methodological problem is very common but often neglected in prejudice research [3, 11].

Therefore, the aim of the current study is twofold. First, we try to replicate DeSteno et al.’s [7] results showing that anger, rather than sadness, creates automatic intergroup bias. We replicate DeSteno et al.’s [7] Study 2 that used the IAT as the measure of intergroup bias, because these authors argued that “compared with priming techniques, the IAT may be more sensitive to individual and group differences and somewhat more reliable across time” (p. 322), which seems reasonable as the results of this experiment have also been replicated in another study of the same research team ([6], Study 1). Second, we aim to test whether this intergroup bias manifests as outgroup derogation as DeSteno et al. hypothesized. In order to do this, we adopted a paradigm called intergroup prisoner’s dilemma-maximizing difference (IPD-MD) that can successfully disentangle ingroup favoritism and outgroup derogation [9]. In this paradigm, two different groups are involved, and each group member is given a monetary endowment that could be allocated to either a within-group pool or a between-group pool. The within-group pool only benefits ingroups, whereas the between-group pool not only benefits ingroups but also harms outgroups. Contributions made to these two pools are indicators on ingroup favoritism and outgroup derogation, respectively. Unlike Halevy et al.’s [9] original task in which participants interacted with real group members, we used a revised version of IPD-MD in which participants were led to believe they were connected to other players through the internet, thus creating fictitious interactions between members.

## Methods

### Participants

In total, 99 college students from a Swedish University (69 women; 91 Whites; mean age = 24.79 years, *SD* = 5.19) were recruited. In return each of them received a supermarket gift card valued at 50 SEK (approximately $6). This study was carried out in accordance with the recommendation of experimental guidelines of Lund University with written informed consent from all subjects. Because of the informed consent and the hypothetical and non-intrusive nature of the study, no formal ethics committee was required to review the study as per the university’s guidelines and national regulations.

### Procedure

The procedure was similar to DeSteno et al.’s [7] Study 2. First, all participant went through the minimal group categorization procedure and were assigned to a specific group. Next, they performed the IAT task for measuring automatic intergroup attitudes. The manipulation of emotion was embedded into the IAT task, as in DeSteno et al.’s [7] study. After the IAT task, participants received the revised IPD-MD task, which was used for disentangling ingroup favoritism and outgroup derogation. Finally, participants reported their explicit attitudes towards ingroups and outgroups as well as their emotional states.

#### Creation of minimal groups

Participants saw some pictures on the computer and answered questions about what they had seen [20]. These pictures were ambiguous and could be interpreted in different ways. After participants submitted answers to all pictures, they were informed that there were two categories of people: “figure-oriented” and “grounding-oriented”. They were categorized as “figure-oriented” based on their replies to these questions, and were then given a wristband with grey color on which the label “figure-oriented” was clearly visible.

#### IAT and emotion manipulation

After the creation of minimal groups, participants were instructed to classify pictures and words. They were informed that pictures with grey background color were individuals with figure-orientation (i.e., ingroups) and pictures with white background color were individuals with grounding-orientation (i.e., outgroups). Twelve pictures (6 males and 6 females) with neutral facial expression were selected from a database of Caucasian faces [15]. Half of these pictures indicated figure-oriented individuals while the other half indicated grounding-oriented individuals (counterbalanced). In all, there were 7 blocks of classification and two of them were critical blocks (i.e., Block 4 and Block 7). In Block 4 participants responded to ingroup pictures and positive words with one key, and to outgroup pictures and negative words with the other key. In Block 7, they responded to outgroup pictures and positive words with one key, and to ingroup pictures and negative words with the other key. The difference in participants’ reaction times (RT) between Block 4 and Block 7 indicates automatic intergroup bias. If DeSteno et al.’s [7] results could be replicated, in the anger condition we would find longer RT in Block 7 than Block 4. The emotion manipulation was embedded among these blocks. Participants were randomly assigned to three emotion conditions (anger, sadness, and neutral) in which they were required to watch emotional video clips and recall past emotional events. The details of the IAT task and the embedded emotion manipulation are described in the Additional file 1.

#### IPD-MD

After the IAT task, participants played an online game in which they believed that they would play with other participants, both figure-oriented and grounding-oriented. In reality, however, they were playing the game by themselves. In this game, six players were connected through the internet. Besides the participant, there were two other figure-oriented players and three grounding-oriented players, which resulted in three players in each group. Participants had ten tokens available that could be allocated to three different pools: the individual pool, the within-group pool, and the between-group pool. For each token they invested in the individual pool, they would only benefit themselves and get 20 SEK. They would benefit both themselves and their ingroup members if they put the tokens into the within-group pool. Each of them would get 10 SEK for each token. Finally, if the token was invested in the between-group pool, it would not only benefit each of the ingroup members, but also have a negative effect on outgroups. For each token, all ingroup members would get 10 SEK, but all outgroup members would lose 10 SEK at the same time.

#### Explicit intergroup attitudes

Participants then evaluated the ingroup and the outgroup separately on five dimensions (unintelligent-intelligent, bad-good, unpleasant-pleasant, dishonest-honest, awful-nice) by using a 9-point scale. Their responses were then averaged for both the ingroup (α = .93) and the outgroup (α = .91).

#### Emotional states

At the end of the experiment, seven items were used to assess participants’ emotional states: 1) How sad do you feel now? 2) How depressed do you feel now? 3) How down are you now? 4) How angry do you feel now? 5) How annoyed are you now? 6) How frustrated are you now? 7) How irritated are you now? Participants responded on a 9-point rating scale ranging from “not at all” to “very much”.

## Results

### Manipulation check

One-way ANOVA with the seven items assessing emotional states as dependent measures showed that only scores on the anger item (i.e., “How angry do you feel now?”) differ significantly between conditions, *F* (2, 96) = 5.78, *p* = .004. Subsequent post hoc test (LSD) showed that participants in the anger condition (*M =* 3.58, *SD =* 2.39) reported higher anger than those in the sadness condition (*M =* 2.19, *SD =* 2.02), *p* = .007, as well as those in the neutral condition (*M =* 2.03, *SD =* 1.64), *p* = .002. For the sadness item (i.e., “How sad do you feel now”), although participants tended to report higher sadness in the sadness condition (*M =* 3.66, *SD =* 2.42) than in the anger condition (*M =* 3.27, *SD =* 1.92) and the neutral condition (*M =* 3.21, *SD =* 2.14), these differences were not significant, *F* (2, 96) = 0.41, *p* = .665. Therefore, it seems that our manipulation of anger was successful, but the manipulation of sadness was not as powerful as expected.

### IAT analyses

**Fig. 1:**
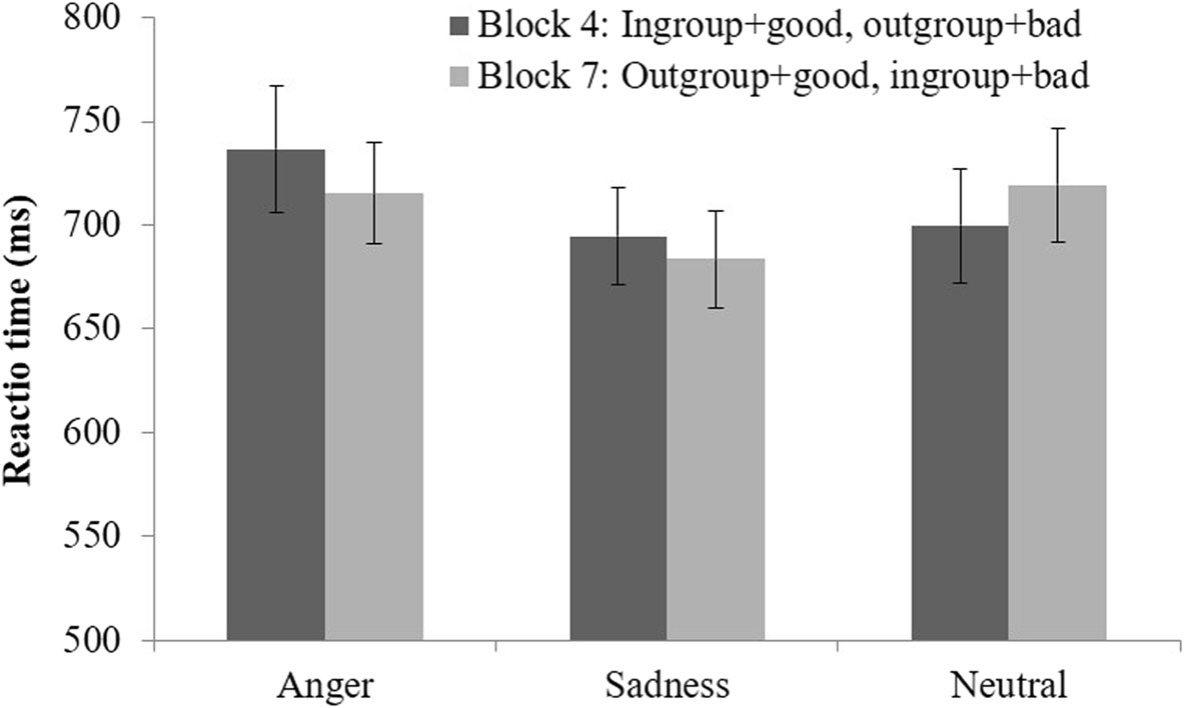
Reaction time in the IAT task as a function of emotion and stimulus pairing. Error bars indicate standard errors

A 3 (condition: sadness vs. anger vs. neutral) by 2 (IAT block: Block 4 vs. Block7) ANOVA with repeated measure on the latter factor showed no significant results. As depicted in Fig. 1, the main effect of condition, *F* (2, 96) = 0.61, *p* = .548, the main effect of IAT block, *F* (1, 96) = 0.11, *p* = .742, and the interaction, *F* (2, 96) = 0.89, *p* = .413, were all far from significance. Therefore, DeSteno et al.’s [7] results were not replicated.

Further, in each condition, we used Bayes factor analysis to examine whether the null hypothesis (i.e., RT in Block 4 is not different RT in Block 7) was more likely given the current data. The traditional significance testing can only provide information regarding whether H_0_ (the null hypothesis) can be rejected. However, rejecting H_0_ does not necessarily mean there is no difference between conditions. The Bayes factor provides a continuous measure of evidence for H_1_ (the alternative hypothesis) over H_0_ [8]. When the Bayes factor is 1, the data favors neither H_1_ nor H_0_. As the Bayes factor increases above 1 (toward infinity), the data favors H_1_ over H_0_. On the contrary, the data favors H_0_ over H_1_ as the Bayes factor decreases below 1 (toward 0). Researchers suggest that a Bayes factor (*BF*_10_) that is bigger than 3 would provide substantial support for H_1_ whereas a Bayes factor (*BF*_10_) smaller than 1/3 would provide substantial support for H_0_. Our results supported the null hypothesis in all conditions (for anger, *BF*_10_ = 0.27; for sadness, *BF*_10_ = 0.22; for neutral, *BF*_10_ = 0.25).

### IPD-MD

**Fig. 2:**
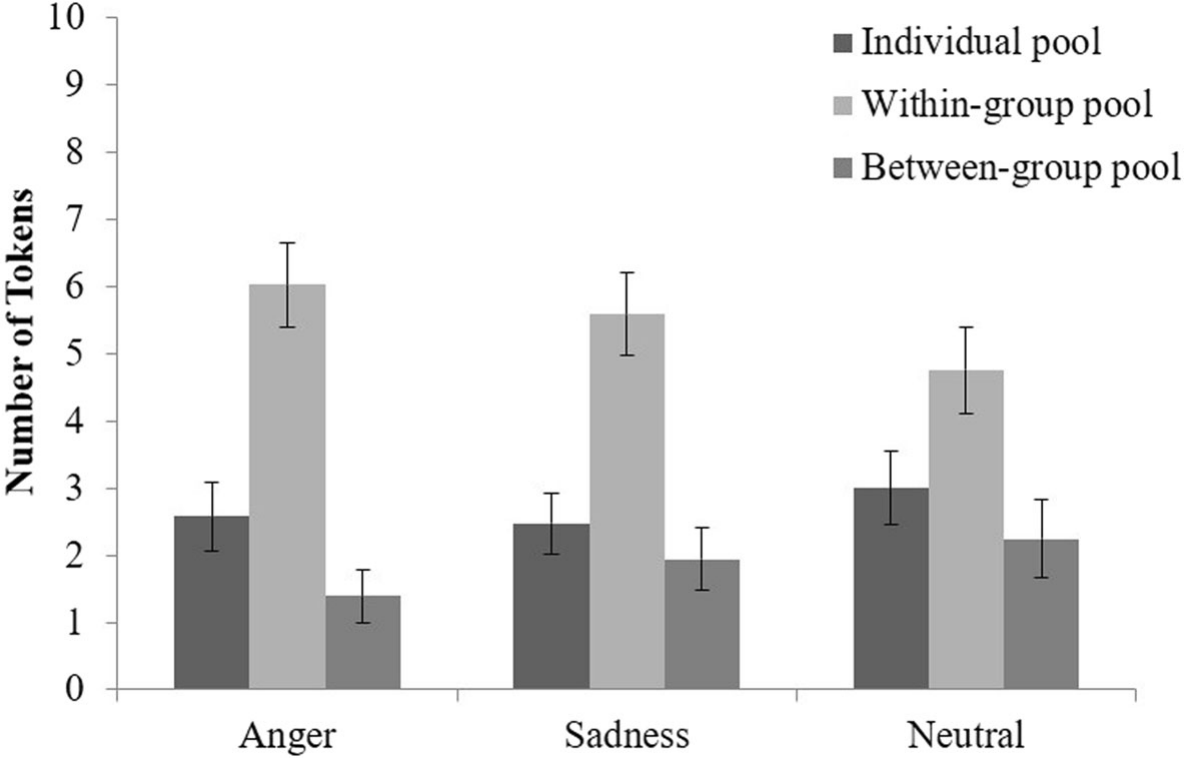
Tokens allocated to each pool as a function of emotion. Error bars indicate standard errors

A 3 (condition: anger vs. sadness vs. neutral) by 3 (pool: individual vs. within-group vs. between-group) ANOVA with repeated measure on the latter factor showed a significant main effect of pool, *F* (2, 192) = 24.04, *p* < .001, partial *η*^2^ = .20, as shown in Fig. 2. However, both the main effect of condition, *F* (2, 96) = 0.38, *p* = .688, and the interaction, *F* (4, 192) = 0.77, *p* = .547, were not significant. Post hoc test of the main effect of pool (LSD) showed that participants invested more in the within-group pool (*M =* 5.45, *SD =* 3.61) than the individual pool (*M =* 2.69, *SD =* 2.89, *p* < .001) and the between-group pool (*M =* 1.86, *SD =* 2.81, *p* < .001). They also allocated slightly more to the individual pool than the between-group pool, *p* = .065. These results indicate that participants in all of the three conditions showed ingroup favoritism rather than outgroup derogation.

### Explicit attitudes

**Fig. 3:**
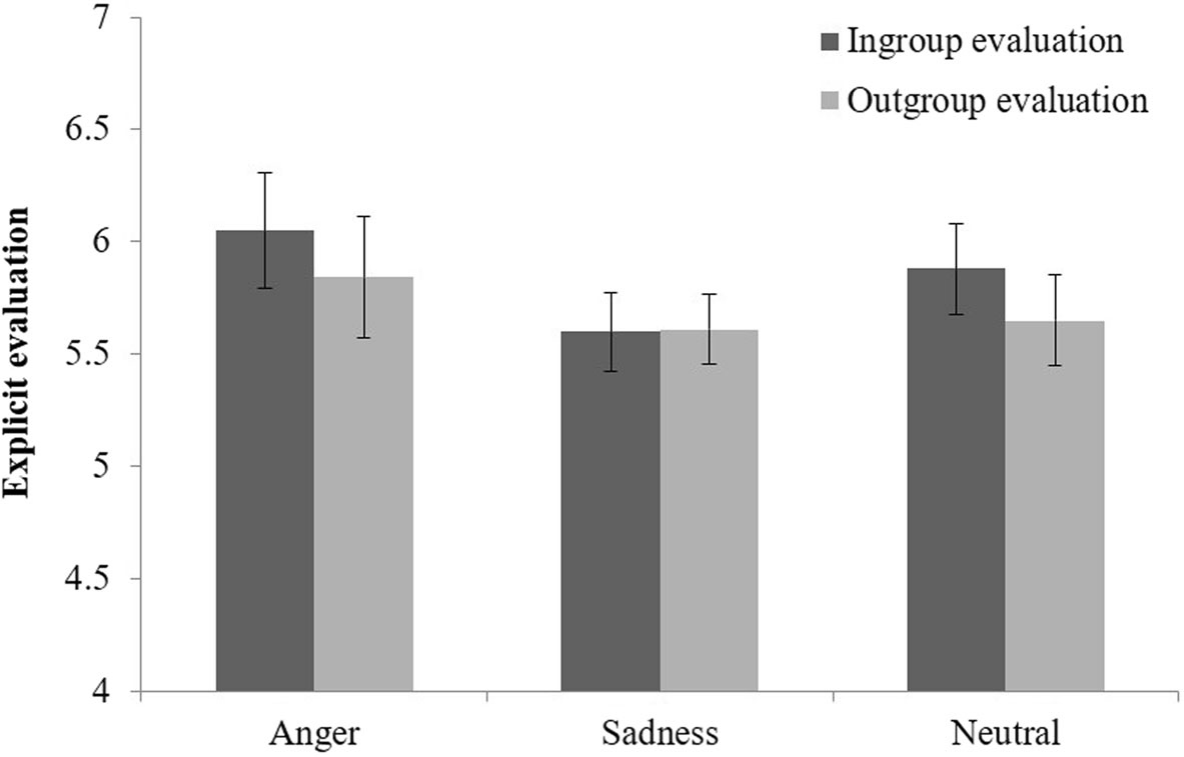
Explicit attitudes toward the ingroup and the outgroup as a function of emotion. Error bars indicate standard errors

A 3 (condition: anger vs. sadness vs. neutral) by 2 (group: ingroup evaluation vs. outgroup evaluation) ANOVA with repeated measure on the latter factor showed only a marginally significant main effect of group, *F* (1, 96) = 3.30, *p* = .073, partial *η*^2^ = .03, as shown in Fig. 3, which suggests that participants evaluated the ingroup (*M =* 5.85, *SD =* 1.23) more positively than the outgroup (*M =* 5.70, *SD =* 1.19). The main effect of condition, *F* (1, 96) = 0.71, *p* = .496, and the interaction, *F* (2, 96) = 0.96, *p* = .386, were far from significance. Interestingly, the evaluation of the outgroup was above the midpoint of the scale (i.e., “5”), *t* (98) = 5.82, *p* < .001, which showed no sign of outgroup derogation. This pattern held in all of the three emotion conditions (for anger, *t* (32) = 3.10, *p* = .004; for sadness, *t* (31) = 3.95, *p* < .001; for neutral, *t* (33) = 3.51, *p* = .001).

## Discussion

The current experiment failed to replicate DeSteno et al.’s [7] results showing that anger, rather than sadness, created automatic intergroup bias as measured by the IAT task. Because there are several differences between DeSteno et al.’s [7] procedure and the current replication’s procedure, here we list these differences and analyze which of them may be related to the replication failure.

First, regarding creation of minimal groups, DeSteno et al. used an estimation task in which participants estimated the frequency of various events and then were randomly assigned as either an “overestimator” or an “underestimator”. The current replication used a perceptual task in which participants categorized ambiguous pictures and were randomly assigned as either a “figure-oriented” person or a “grounding-oriented” person. This difference should not the cause of the replication failure because both tasks have been verified in the literature. The explicit intergroup evaluation also showed more positive attitudes toward the ingroup than the outgroup in both DeSteno et al.’s [7] Study 2 and the current replication, suggesting the manipulation of creating minimal groups was successful in both studies.

Second, to ensure that participants remembered their group membership, the current replication required participants to wear a grey wristband on which the group identity was clearly visible. This method was neutral and unlikely to introduce confounding factors. By contrast, DeSteno et al. asked their participants to wear colored wristbands (red and blue). Considering the impact of high-contrast colors, especially red, on human perception such as aggression (e.g., [10]), we cannot rule out the possibility that DeSteno et al.’s results were artifacts caused by this irrelevant factor (i.e., colored wristbands).

Third, regarding emotional induction, in addition to the autobiographical memory method used by DeSteno et al. [7], we also used emotional video clips, which has been demonstrated to be the most effective way of inducing emotions in the lab [12, 21]. The manipulation check also showed that participants in the anger condition expressed more anger than those in the sadness and the neutral conditions. Therefore, the replication failure regarding the focal hypothesis that anger creates automatic intergroup bias should not be attributed to the emotional induction procedure. However, the manipulation check suggested that our manipulation of sadness was not successful due to unknown reasons. Therefore, it is difficult for our data to provide clues regarding whether sadness could create automatic intergroup bias or not.

Previous findings regarding the influence of emotions on intergroup attitudes in real contexts might shed light on the failure of the current replication. For example, Dasgupta et al. [6] found that anger only increased bias against anger-relevant groups (e.g., Arabs) but not against other groups, while disgust only increased bias against disgust-relevant groups (e.g., homosexuals) but not against other groups. Their rationale was that emotions could increase implicit prejudice only if the induced emotion is applicable to the outgroup stereotype. In a minimal group context, however, there was no well-established outgroup stereotype. Therefore, the influence of emotions should not be stable in a minimal group context. Because this is the first attempt of replicating DeSteno et al.’s [7] findings and we only replicated their Study 2, we look forward to more replications on this topic before drawing any strong conclusion.

Results of the IPD-MD showed that participants across all emotion conditions exhibited high level of ingroup favoritism. This is intriguing because they, at a cost of their personal interest, benefit ingroup members they do not have any information except for the shared perceptual style. In contrast, there is little evidence that they tend to harm outgroups. The explicit intergroup evaluation also revealed the same pattern. These results suggest that, when there is no competition or conflict between groups, individuals, even in a bad emotional state such as anger, generally show ingroup love rather than outgroup hate. However, anger might instigate outgroup derogation when intergroup competition or conflict is involved. This is in need of future research.

## Conclusion

Although previous studies showed that anger created automatic intergroup bias in a minimal group context, the current research failed to replicate this effect. Further, by using the IPD-MD paradigm, the current research found that participants across all emotion conditions exhibited high level of ingroup favoritism, but there was little evidence of outgroup derogation. These results suggest that, when there is no competition or conflict between groups, individuals, even in a bad emotional state such as anger, generally show ingroup love rather than outgroup hate.

## Supplementary information

**Additional file 1.**

## Data Availability

The data file is available on Open Science Framework at https://osf.io/db8ag/?view_only=78c9fe90824449a5ba49e659eb645ef2.

## Abbreviations

IAT: Implicit Association Test
IPD-MD: Intergroup prisoner’s dilemma-maximizing difference
RT: Reaction times

## References

[1] Amodio DM, Hamilton HK (2012). Intergroup anxiety effects on implicit racial evaluation and stereotyping. Emotion.

[2] Bodenhausen GV, Mussweiler T, Gabriel S, Moreno KN, Forgas JP (2001). Affective influences on stereotyping and intergroup relations. Handbook of affect and social cognition.

[3] Brewer MB (2007). The importance of being we: human nature and intergroup relations. Am Psychol.

[4] Bukowski M, Dragon P (2014). The impact of incidental fear and anger on in- and outgroup attitudes. Pol Psychol Bull.

[5] Conrey FR, Sherman JW, Gawronski B, Hugenberg K, Groom CJ (2005). Separating multiple processes in implicit social cognition: the quad model of implicit task performance. J Pers Soc Psychol.

[6] Dasgupta N, Desteno D, Williams LA, Hunsinger M (2009). Fanning the flames of prejudice: the influence of specific incidental emotions on implicit prejudice. Emotion.

[7] DeSteno D, Dasgupta N, Bartlett MY, Cajdric A (2004). Prejudice from thin air: the effect of emotion on automatic intergroup attitudes. Psychol Sci.

[8] Dienes Z, Mclatchie N (2018). Four reasons to prefer Bayesian analyses over significance testing. Psychon Bull Rev.

[9] Halevy N, Bornstein G, Sagiv L (2008). “In-group love” and “out-group hate” as motives for individual participation in intergroup conflict: a new game paradigm. Psychol Sci.

[10] Hill RA, Barton RA (2005). Red enhances human performance in contests. Nature.

[11] Hewstone M, Rubin M, Willis H (2002). Intergroup bias. Annu Rev Psychol.

[12] Joseph DL, Chan MY, Heintzelman SJ, Tay L, Diener E, Scotney VS (2020). The manipulation of affect: a meta-analysis of affect induction procedures. Psychol Bull.

[13] Kossowska M, Bukowski M, Van Hiel A (2008). The impact of submissive versus dominant authoritarianism and negative emotions on prejudice. Personal Individ Differ.

[14] Kuppens T, Pollet TV, Teixeira CP, Demoulin S, Roberts SC, Little AC (2012). Emotions in context: anger causes ethnic bias but not gender bias in men but not women. Eur J Soc Psychol.

[15] Langner O, Dotsch R, Bijlstra G, Wigboldus DHJ, Hawk ST, van Knippenberg A (2010). Presentation and validation of the Radboud faces database. Cognit Emot.

[16] Lench HC, Flores SA, Bench SW (2011). Discrete emotions predict changes in cognition, judgment, experience, behavior, and physiology: a meta-analysis of experimental emotion elicitations. Psychol Bull.

[17] Loewenstein GF, Weber EU, Hsee CK, Welch N (2001). Risk as feelings. Psychol Bull.

[18] Nosek BA, Lakens D (2014). Registered reports: a method to increase the credibility of published results. Soc Psychol.

[19] Open Science Collaboration (2015). Estimating the reproducibility of psychological science. Science.

[20] Otten S, Moskowitz GB (2000). Evidence for implicit evaluative in-group bias: affect-biased spontaneous trait inference in a minimal group paradigm. J Exp Soc Psychol.

[21] Westermann R, Spies K, Stahl G, Hesse FW (1996). Relative effectiveness and validity of mood induction procedures: a meta-analysis. Eur J Soc Psychol.

