# Supplementary material for: Is there prejudice from thin air? Replicating the effect of emotion on automatic intergroup attitudes
Source: BMC Psychol. 2020 May 6;8:47. doi: 10.1186/s40359-020-00414-4 (PMC7201959; doi:10.1186/s40359-020-00414-4)
Supplement: Supplementary file 1 — Additional file 1. [file 40359_2020_414_MOESM1_ESM.docx]

**Additional file 1**

In Block 1 of the IAT task, participants pressed “f” and “j” when they saw figure-oriented individuals and grounding-oriented individuals respectively (24 trials). In Block 2 they pressed “f” and “j” when they saw positive words and negative words respectively (24 trials). Before continuing with the following IAT blocks, participants were randomly assigned to three emotion conditions in which they watched a short video clip lasting for 2 minutes and recalled past events for 3 minutes. The clip was from the movie “My bodyguard” in the anger condition and from the “The Champ” in the sadness condition, respectively (Gross, Robert, & Levenson, 1995). Participants were then asked to list five events that made them very angry or sad during the past one year. In the neutral condition, the clip showed only color bars (Gross et al., 1995) and participants were then asked to list five events they were doing regularly during the past one year.

After the recall process, participants continued with the following IAT blocks. In Block 3 (24 trials) and Block 4 (48 trials), they responded to both pictures and words. They were asked to press “f” when they saw figure-oriented individuals or positive words and to press “j” when they saw grounding-oriented individuals or negative words. Block 3 was a practice block, whereas Block 4 was the critical block.

In Block 5, participants classified the same pictures but the instruction was changed. They needed to press “f” when they saw grounding-oriented individuals and to press “j” when they saw figure-oriented individuals (24 trials). After this, they received the emotion manipulation again by watching a 2-minute clip and engaging in 3-minute recalling. The clip was from the movie “Cry Freedom” in the anger condition, from the movie “John Q” in the sadness condition, and from Gross et al.’s (1995) materials showing geometric lines, respectively. Participants were then asked to choose one event they wrote after the first video clip and describe it in detail.

Following the second emotion induction, in Block 6 (24 practice trials) and Block 7 (48 critical trials) of the IAT task, participants responded to both pictures and words. They were informed to press “f” when they saw grounding-oriented individuals or positive words, and to press “j” when they saw figure-oriented individuals or negative words. During all blocks participants were asked to respond as quick and accurate as possible.

**References**

Gross, J. J., & Levenson, R. W. (1995). Emotion elicitation using films. *Cognition & emotion*, *9*, 87-108. doi:10.1080/02699939508408966
